# SaE-GBLS: an effective self-adaptive evolutionary optimized graph-broad model for EEG-based automatic epileptic seizure detection

**DOI:** 10.3389/fncom.2024.1379368

**Published:** 2024-07-11

**Authors:** Liming Cheng, Jiaqi Xiong, Junwei Duan, Yuhang Zhang, Chun Chen, Jingxin Zhong, Zhiguo Zhou, Yujuan Quan

**Affiliations:** ^1^Department of Cerebral Function, The Second Affiliated Hospital of Guangzhou University of Chinese Medicine, Guangzhou, China; ^2^College of Information Science and Technology, Jinan University, Guangzhou, China; ^3^Faculty of Data Science, City University of Macau, Macao, Macao SAR, China; ^4^Guangdong Provincial Key Laboratory of Traditional Chinese Medicine Informatization, Jinan University, Guangzhou, China; ^5^College of Engineering and Computer Science, Australian National University, Canberra, ACT, Australia; ^6^School of Information and Electronics, Beijing Institute of Technology, Beijing, China

**Keywords:** self-adaptive evolutionary algorithm, graph regularized broad learning system, EEG, seizure detection, epilepsy

## Abstract

**Introduction:**

Epilepsy is a common neurological condition that affects a large number of individuals worldwide. One of the primary challenges in epilepsy is the accurate and timely detection of seizure. Recently, the graph regularized broad learning system (GBLS) has achieved superior performance improvement with its flat structure and less time-consuming training process compared to deep neural networks. Nevertheless, the number of feature and enhancement nodes in GBLS is predetermined. These node settings are also randomly selected and remain unchanged throughout the training process. The characteristic of randomness is thus more easier to make non-optimal nodes generate, which cannot contribute significantly to solving the optimization problem.

**Methods:**

To obtain more optimal nodes for optimization and achieve superior automatic detection performance, we propose a novel broad neural network named self-adaptive evolutionary graph regularized broad learning system (SaE-GBLS). Self-adaptive evolutionary algorithm, which can construct mutation strategies in the strategy pool based on the experience of producing solutions for selecting network parameters, is incorporated into SaE-GBLS model for optimizing the node parameters. The epilepsy seizure is automatic detected by our proposed SaE-GBLS model based on three publicly available EEG datasets and one private clinical EEG dataset.

**Results and discussion:**

The experimental results indicate that our suggested strategy has the potential to perform as well as current machine learning approaches.

## 1 Introduction

A neurological condition known as epilepsy is characterized by recurring, unprovoked seizures. An epileptic seizure is described by the international league against epilepsy (ILAE) as “a passing event characterized by indications and/or symptoms resulting from abnormal, excessive, or synchronous neuronal activity in the brain.” The world health organization estimates that epilepsy affects about 50 million individuals globally, and at least 100 million individuals experience the effects of this disorder at least once in their lifetime (Alarcón and Valentín, 2012). Seizures typically last from seconds to a few minutes and can happen unexpectedly without any warning signs, leading to serious injuries such as fractures, burns, and occasionally even death (Hannah and Brodie, 1998). Seizure period detection is a crucial aspect of diagnosing, treating, and researching epilepsy. Accurate detection of seizure periods is crucial for determining the frequency, duration, and characteristics of seizures, as well as monitoring responses to treatment and predicting outcomes. Electroencephalogram (EEG) is a technique that uses multiple electrodes placed on the subject's head, based on specific criteria, to record neural electrophysiological brain activity. Various studies have utilized techniques such as electrocorticography (ECoG), magnetoencephalography (MEG), single-photon emission computed tomography (SPECT), stereo electroencephalography (SEEG), positron emission tomography (PET), and magnetic resonance imaging (MRI) for epilepsy monitoring. EEG is a non-invasive, portable, and cost-effective method widely used in epilepsy research compared to other methods.

Machine learning has been continuously developed and applied in various fields. In recent years, there has been significant interest in using machine learning techniques for disease diagnosis. This is because these techniques have the potential to save a significant amount of medical resources. Various machine learning techniques have been used for epilepsy, such as KNN (Amin et al., 2015; Shih et al., 2022), SVM (Chen et al., 2017, 2020; Shih et al., 2022), decision trees (Siddiqui et al., 2019; Shih et al., 2022), Naïve Bayes (Shih et al., 2022), and logistic regression (Shih et al., 2022). Machine learning-based methods are being used for the detection of epilepsy seizures. Including Random Forest (Sharma et al., 2018; Siddiqui et al., 2019) and Boosting (Siddiqui et al., 2019). Deep learning-based methods have been utilized in epilepsy research alongside machine learning techniques. In comparison to traditional machine learning, deep learning offers stronger learning ability, improved adaptability, and multiple layers of processing that can handle various levels of abstract data representation. For instance, EEGNet, a compact convolutional neural network utilized for brain-computer interfaces based on EEG, was developed in Lawhern et al. (2018). Sui et al. (2019) introduced a novel method to distinguish between focal and non-focal intracranial electroencephalogram (iEEG) signals using the short-time fourier transform (STFT) and convolutional neural networks (CNN).

While deep neural networks have achieved remarkable advancements in various applications, their complexity and the need to calibrate numerous parameters present a challenge. Deep neural network training is time-consuming and requires substantial computing resources. Additionally, existing seizure detection models often face overfitting issues due to the limited size of the dataset, which leads to low accuracy. Therefore, to compensate for the limitations of deep learning networks, a novel technique called the random vector function connection neural network (RVFLNN) and broad learning system (BLS) is proposed in Chen and Liu (2017). This technique aims to prevent the need for network retraining and enable rapid network reconstruction. A graph mosaic-width learning model is proposed in this study. The model incorporates a graph regularization term into the standard broad learning model. Additionally, the training data is used to generate the intrinsic graph and the penalty graph. The fundamental geometric structure of the data is still considered, and the features that can be quickly reconstructed using BLS are still preserved. However, the number of feature nodes and enhancement nodes is predetermined, and the parameters of these nodes are randomly generated and utilized during training. This will result in numerous suboptimal nodes in the GBLS network. As a popular method for selecting network parameters, the self-adaptive evolutionary algorithm continuously updates the strategy pool based on the selection experience of candidate solutions. Ultimately, it selects the optimal strategy from the strategy pool. This paper proposes optimizing the characteristic node parameters and enhanced node parameters using a self-adaptive evolutionary algorithm. The parameters of the characteristic nodes and enhanced nodes are treated as evolutionary individuals, and the process of “mutation-cross-selection” is continuously performed until the stopping conditions are met. The optimal node parameters are substituted into the graph-regularized generalized learning system to train the model. Finally, seizure detection in epilepsy is performed using a trained model. The following list summarizes the significant contributions of this paper.

- The present study introduces a novel self-adaptive evolutionary graph-regularized broad learning system (SaE-GBLS) by incorporating an effective optimization algorithm. This system preserves the quick reconstruction capability of BLS while considering the intrinsic geometric structure of the data and determining the optimal number of nodes.
- To the best of our knowledge, this is the first instance where SaE-GBLS has been utilized for seizure detection, and our proposed approach demonstrates competitive performance.
- Three publicly available epilepsy datasets and a set of private clinical data were utilized to assess the validity of our approach.

The organization of this paper is as follows: Section 2 provides a concise overview of the classic broad learning system, graph regularized broad learning system, and differential evolution. In Section 3, we introduce SaE-GBLS along with comprehensive details of our proposed approach for detecting epileptic seizures. Section 4 focuses on evaluating the performance of our approach through multiple experiments, providing detailed results and analysis. Finally, Section 5 concludes the paper by summarizing the key findings and contributions.

## 2 Preliminaries

### 2.1 Broad learning system

BLS is a flat network structure based on RVFLNN. Unlike traditional deep learning networks, the incremental learning algorithm in BLS enables it to have efficient and fast reconstruction capabilities. This avoids the time-consuming phenomenon caused by a large number of hyperparameters in the deep learning framework. BLS utilizes pseudo-inverse and ridge regression methods to efficiently calculate weights that connect feature nodes and enhancement nodes to the output parameters.

Suppose the input samples are *X, Y*, where *X* ∈ ℝ^*N*×*M*^, *Y* ∈ ℝ^*N*×*C*^ and *N* is the number of samples, *M* is the dimension of *X*, *C* is the dimension of *Y*. Using the feature mapping function ξ_*i*_(*XW*_*z*_*i*__ + α_*z*_*i*__) to map the sample data and result mapping feature *Z*_*i*_. Where *W*_*z*_*i*__ and α_*z*_*i*__ are the parameters of *Z*_*i*_. Different feature mapping functions can be different, and the resulting mapping feature set is $Z^{n}\equiv \left[Z_{1},\ldots ,Z_{n}\right]\in {\mathbb{R}}^{N\times nk}$, *nk* is the number of feature mappings. Pass all feature nodes through enhancement functions $\eta_{j}\left(Z^{n}W_{h_{j}}+\gamma_{h_{j}}\right)$ to get the enhancement nodes *H*_*j*_ and its parameters are *W*_*h*_*j*__ and γ_*h*_*j*__. The collection of all enhanced nodes is $H^{m}\equiv \left[H_{1},\ldots ,H_{m}\right]\in {\mathbb{R}}^{N\times m}$ where *m* is the number of enhancement nodes. Combine the feature nodes set and the enhanced nodes set to obtain a direct connection to the output node *A* = [*Z*^*n*^|*H*^*m*^].

The broad learning model is expressed by the following Equations (1) and (2):


(1) $$\begin{array}{l} Ŷ=\left[Z_{1},Z_{2},\ldots ,Z_{n}|H_{1},H_{2},\ldots ,H_{m}\right]W \end{array}$$

(2) $$\begin{array}{l} =\left[Z^{n}|H^{m}\right]W \end{array}$$

**Figure 1:**
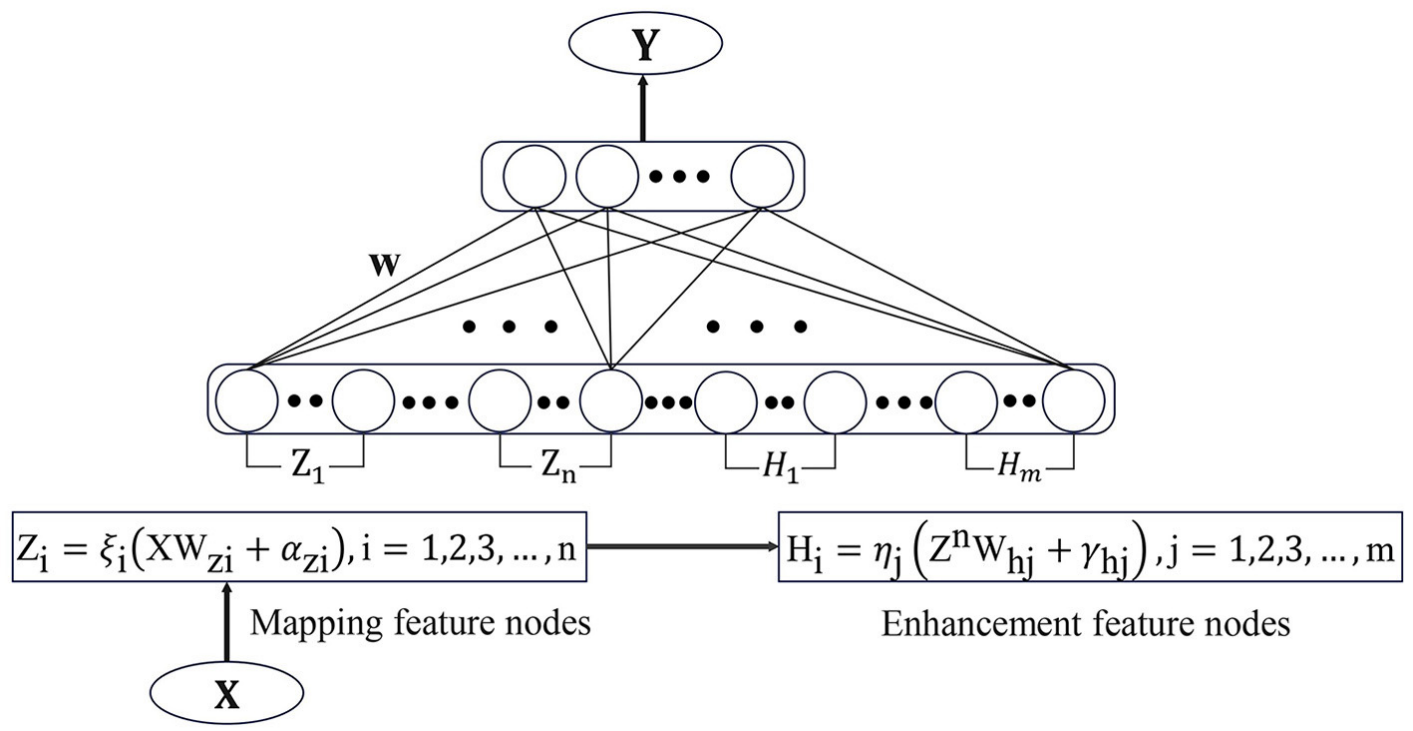
The structure of BLS.

Thus through the ridge regression of [*Z*^*n*^|*H*^*m*^]^+^ we can obtain the output weight


(3) $$\begin{array}{l} W={\left(A^{T}A+\lambda I\right)}^{-1}A^{T}Y \end{array}$$

and λ is a regularization parameter. Figure 1 shows the structure diagram of the standard broad learning system, in which the spare nodes of the model can be quickly established by only calculating the pseudo-inverse of the added nodes.

### 2.2 Graph regularized BLS

In BLS, by minimizing the Equation (4)


(4) $$\begin{array}{l} arg\;min_{W}J_{BLS}=||Y-AW|{|}^{2}+\lambda ||W|{|}^{2}, \end{array}$$

we obtain the expression for the output (3). Although the BLS model can be solved quickly, it does not take into account the geometric structure of the underlying data. Based on the basic BLS structure, Jin et al. (2018) proposed graph regularized BLS (GBLS). Experiments have proven that the effect of IPGBLS is superior to GBLS in most cases (Jin et al., 2018), therefore IPGBLS is utilized in this article. Add the graph regularization *E*_*G*_ that represents the difference between the local structure of the data into the objective function of GBLS. The objective function is as follow:


(5) $$\begin{array}{l} arg\;min_{W}J_{GBLS}=||Y-AW|{|}^{2}+\lambda_{1}E_{G}+\lambda_{2}||W|{|}^{2} \end{array}$$

and λ_1_, λ_2_ are parameters in the objective function (5) to decide which part is more important. Here, λ_1_ and λ_2_ are weight parameters used to adjust the significance of the EG term and the ||*W*||^2^ term, respectively. GBLS exhibits a high degree of tolerance to the choice of λ_1_ and λ_2_, with optimal values obtained when λ_1_ is relatively large. This suggests that the graph regularization term significantly enhances the BLS model.

The manifold regularization method is incorporated into the optimization process, enhancing the classification ability of GBLS compared to BLS. In one of the GBLS models, specifically IPGBLS, the intrinsic graph *G*^*w*^(*X, V*^*w*^) and penalty graph *G*^*p*^(*X, V*^*p*^) are added simultaneously. The vertex matrix of the intrinsic graph *G*^*w*^ is constructed in Equation (6)


(6) $$\begin{array}{l} V_{ij}^{w}=\{\begin{array}{c} 1,\text{if }l\left(x_{i}\right)=l\left(x_{j}\right)\text{, and }x_{i}\in N_{k_{1}}\left(x_{j}\right),\\ 1,\text{if }l\left(x_{i}\right)=l\left(x_{j}\right)\text{, and }x_{j}\in N_{k_{1}}\left(x_{i}\right),\\ 0,\text{otherwise}. \end{array} \end{array}$$

where $N_{k_{1}}$ is the *k*_1_ neighbor set of *x*_*i*_ and *k*_1_ represents the neighbors' number. The intrinsic graph's Laplacian matrix is *Lw* = *Dw* − *Vw*, where *Dw* = *sum*_*j*_*V*_*i*_*jw*. Consequently, the intrinsic graph's geometric structure can be shown as follows:


(7) $$\begin{array}{l} \underset{ij}{\sum }V_{ij}^{w}||{ŷ}_{i}-{ŷ}_{j}|{|}^{2}=Tr\left({Ŷ}^{T}L^{w}Ŷ\right) \end{array}$$

The penalty graph *G*^*p*^ is the same as *G*^*w*^, the vertex matrix of *G*^*p*^ is shown in Equation (8):


(8) $$\begin{array}{l} V_{ij}^{p}=\{\begin{array}{c} 1,\text{if }l\left(x_{i}\right)\neq l\left(x_{j}\right)\text{, and }x_{i}\in N_{k_{2}}\left(x_{j}\right),\\ 1,\text{if }l\left(x_{i}\right)\neq l\left(x_{j}\right)\text{, and }x_{j}\in N_{k_{2}}\left(x_{i}\right),\\ 0,\text{otherwise}. \end{array} \end{array}$$

and $N_{k_{2}}$ is the *k*_2_ neighbor set of *x*_*i*_ and *k*_2_ is the neighbors' number. The penalty graph's Laplacian matrix is *Lp* = *Dp* − *Vp*, where *Dp* = *sum*_*j*_*V*_*i*_*jp*. As a result, the penalty graph's geometric structure may be shown as follows:


(9) $$\begin{array}{l} \underset{ij}{\sum }V_{ij}^{p}||{ŷ}_{i}-{ŷ}_{j}|{|}^{2}=Tr\left({Ŷ}^{T}L^{p}Ŷ\right) \end{array}$$

In order to make the model has the best classification effect, minimizing the data points of similar geometric structures, that is, minimizing Equation (7). At the same time, maximizing the distance between samples of different structures Equation (9). Thus, the objective is shown in Equation (10)


(10) $$\begin{array}{l} arg\;min\;Tr\left({Ŷ}^{T}{\left({\left(L^{p}\right)}^{-1/2}\right)}^{T}L^{w}\left({\left(L^{p}\right)}^{-1/2}\right)Ŷ\right) \end{array}$$

To ensure the *L*^*p*^ reversible, add a small enough disturbance ζ on the diagonal of *L*^*p*^ that does not affect the model solution. Defined the $L_{IPGBLS}≜{\left({\left(L^{p}\right)}^{-1/2}\right)}^{T}L^{w}\left({\left(L^{p}\right)}^{-1/2}\right)$. The final objective function of IPGBLS is shown in Equation (11)


(11) $$\begin{array}{l} \begin{array}{c} arg\;min_{W}J_{IPGBLS}=\lambda_{1}Tr\left({Ŷ}^{T}L^{IPGBLS}Ŷ\right)\\ +\lambda_{2}||W|{|}^{2}+||Y-AW|{|}^{2} \end{array} \end{array}$$

Then the output weight of the model is:


(12) $$\begin{array}{l} W={\left(A^{T}A+\lambda_{1}A^{T}L_{IPGBLS}A+\lambda_{2}I\right)}^{-1}A^{T}Y \end{array}$$

### 2.3 Differential evolution

The idea of differential evolution (DE) comes from the earliest genetic algorithm, which explores the optimal solution in space through mutation and crossover processes (Price, 2013). Differential evolution algorithm has a good effect on function optimization (Storn, 1996) and noise reduction (Price et al., 2006). At the same time, the differential evolution algorithm has also been fully applied in industries (Rocca et al., 2011) and other fields. Assuming that the objective function is *f*(θ), in order to minimize the objective function, differential evolution establishes a population of *NP* individuals, where the individual vector is $\theta_{i,G}=\left[\theta_{i,G}^{1},\theta_{i,G}^{2},\ldots ,\theta_{i,G}^{D}\right],i=1,2,\ldots ,NP$.

#### 2.3.1 Initialization

In the differential evolution algorithm, the population is initialized. In order to make the initialized parameters cover the largest possible parameter space, the individual is initialized with the following formula: θ_*i,G*_ = θ_*min*_ + *rand*(0, 1)·(θ_*max*_ − θ_*min*_) where $\theta_{min}=\left[\theta_{min}^{1},\theta_{min}^{2},\ldots ,\theta_{min}^{D}\right]$ and $\theta_{max}=\left[\theta_{max}^{1},\theta_{max}^{2},\ldots ,\theta_{max}^{D}\right]$ are the boundary values of the parameter.

#### 2.3.2 Mutation

Choose a mutually exclusive integer *r*_1_, …, *r*_5_ which are different from *i* in the range of [1, *NP*] as the subscript of the parent vector that chooses to generate the difference vectors. We can choose a positive amplification factor, denoted as *F*, within the range of [0, 2] to control the scaling of the difference vector. Additionally, we can select a control parameter, denoted as *K*, within the range of [0, 1]. Here is a list of the most popular mutation strategies (in short, as St).

St 1: DE/rand/1


$$\begin{array}{l} v_{i,G}=\theta_{r_{1}^{i},G}+F\cdot \left(\theta_{r_{2}^{i},G}-\theta_{r_{3}^{i},G}\right) \end{array}$$

St 2: DE/rand-to-best/2


$$\begin{array}{l} \begin{array}{c} v_{i,G}=\theta_{r_{1}^{i},G}+F\cdot \left(\theta_{best,G}-\theta_{r_{1}^{i},G}\right)+F\cdot \left(\theta_{r_{2}^{i},G}-\theta_{r_{3}^{i},G}\right)\\ +F\cdot \left(\theta_{r_{4}^{i},G}-\theta_{r_{5}^{i},G}\right) \end{array} \end{array}$$

St 3: DE/rand/2


$$\begin{array}{l} v_{i,G}=\theta_{r_{1}^{i},G}+F\cdot \left(\theta_{r_{2}^{i},G}-\theta_{r_{3}^{i}}\right)+F\cdot \left(\theta_{r_{4}^{i}}-\theta_{r_{5}^{i},G}\right) \end{array}$$

St 4: DE/current-to-rand/1


$$\begin{array}{l} v_{i,G}=\theta_{i,G}+K\cdot \left(\theta_{r_{1}^{i},G}-\theta_{i,G}\right)+F\cdot \left(\theta_{r_{2}^{i},G}-\theta_{r_{3}^{i},G}\right) \end{array}$$

“DE/rand/1” is characterized by its strong ability to solve multi-modal problems, yet the findings' convergence takes a long period. The feature of “DE/rand-to-best/2” is that it can achieve very good results in dealing with single-peak problems and can converge quickly. However, when dealing with multimodal problems, this method is prone to getting trapped in local optimal solutions. Compared with “DE/rand/1”, it can produce better disturbances, but it also takes more time to reach convergence. “DE/current-to-rand/1” can also generate more disturbances than one, and has a better effect in dealing with multi-objective optimization problems.

#### 2.3.3 Crossover

After the mutation vector is generated, crossover operation is performed on each mutation vector $v_{i,G}=\left[v_{i,G}^{1},v_{i,G}^{2},\ldots ,v_{i,G}^{D}\right]$ and obtain the trail vector $u_{i,G}=\left[u_{i,G}^{1},u_{i,G}^{2},\ldots ,u_{i,G}^{D}\right]$:


(13) $$\begin{array}{l} u_{i,G}^{j}=\{\begin{array}{c} v_{i,G}^{j},\text{if }\left(rand_{j}\leq CR\right)\text{ or }\left(j=j_{rand}\right),\\ \theta_{i,G}^{j},\text{otherwise} \end{array} \end{array}$$

So that the value of the parameter has more possibilities. Among them, the crossover rate, denoted as *CR*, is selected within the range of [0, 1). A larger crossover rate makes the feasible solution have a larger feasible region. Randomly select an index *j*_*rand*_ between 1 and *D* to ensure that at least one parameter in the newly generated test vector has crossed.

#### 2.3.4 Selection

Finally, select the target vector or trail vector with a smaller objective function value to enter the next generation population, and repeat the “mutation-cross-selection” operation until the stopping condition is met.

## 3 Proposed method

**Figure 2:**
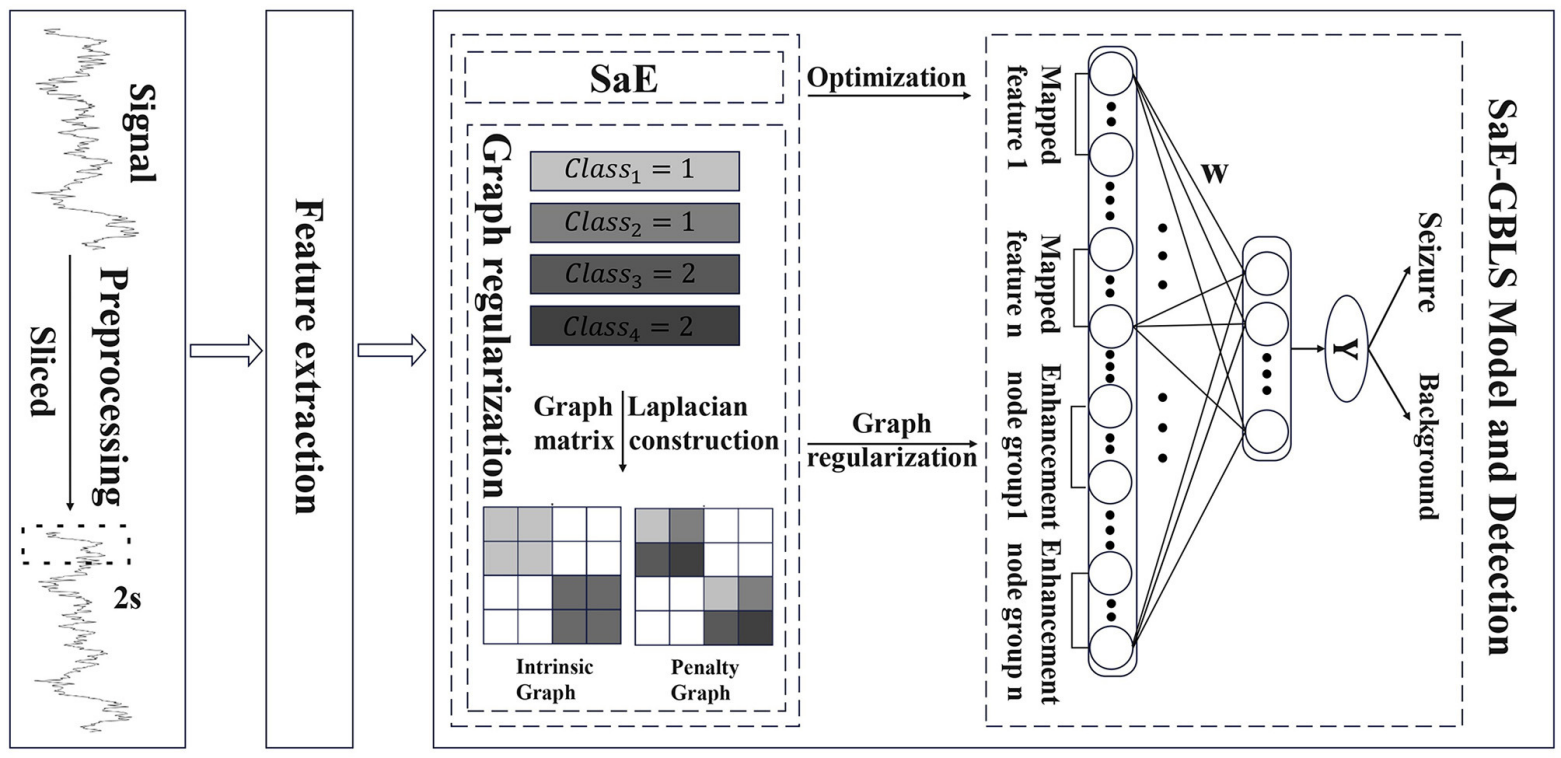
The architecture of SaE-GBLS based automatic epileptic detection.

We propose SaE-GBLS for the classification of epileptic seizure signals. This approach has the potential to enhance our understanding and management of epilepsy, ultimately resulting in improved outcomes for patients. The input to this method is the extracted features from pre-processed EEG signals, aiming to automatically detect and classify epileptic seizure segments. The output is a binary label, where 0 represents non-seizure segments, and 1 indicates seizure segments. The main methods employed are the SaE optimization algorithm and the GBLS classifier. we employed various activation functions, including sigmoid, tanh, tansig, and relu, to obtain richer non-linear feature mappings. Figure 2 illustrates the proposed model.

### 3.1 Preprocessing

Due to inconsistencies in the number of recording channels across subjects in the CHB-MIT and Patient datasets, only subjects with a consistent channel count were selected to ensure uniform feature dimensionality. The EEG signals were segmented into 2-s epochs with an overlap of 0.5 s. This choice of epoch length and overlap ratio was motivated by several considerations. A 2-s window provides a suitable time resolution to capture transient yet meaningful patterns in brain activity without obscuring rapid fluctuations. The 0.5-s overlap was introduced to augment the data and mitigate the potential loss of critical information at segment boundaries, thereby improving the model's ability to capture patterns spanning adjacent epochs. This segmentation approach, with a 2-s epoch length and moderate overlap, is a common practice in EEG signal processing.

### 3.2 Feature extraction

Time domain features are crucial for EEG signal classification as they capture the temporal variations in EEG signals, provide a comprehensive description of the data, are easy to compute and interpret, and are sensitive to changes caused by various factors. Therefore, thirteen features in the time domain are extracted from each channel of both the normal and seizure groups. These features include mean, standard deviation (std), peak-to-peak (p2p), variance (var), minimum (min), maximum (max), argminim, argmaxim, mean-square, root mean square (rms), sum of absolute difference, skewness (skew), and kurtosis (kurt).

### 3.3 Classification

The GBLS model has many non-optimal parameters. In order to mitigate the negative impact of a high percentage of non-optimal nodes on the model's effectiveness, this paper proposes a combination of the adaptive differential evolution algorithm with GBLS.

#### 3.3.1 Initialization

The feature node parameters *Z*_*i*,(*k, G*)_ and enhanced node parameters *H*_*j*,(*k, G*)_ in the GBLS model are connected together as part of the adaptive evolution algorithm. Randomly generate the initial population: *A*_*k,G*_ = [*Z*_1,(*k, G*)_, …, *Z*_*n*,(*k, G*)_, *H*_1,(*k, G*)_, …, *H*_*m*,(*k, G*)_], where *k* = 1, 2, …, *NP* is the index of the individual and *G* is the number of iterations.

#### 3.3.2 Calculate the current optimal solution

Calculate the output weight *W*_*k,G*_ using Equation (12) for each individual vector.calculate the corresponding Root Mean Square Error (RMSE) using the formula $RMSE_{k,G}=\sqrt{\frac{\left||A_{k,G}W_{k,G}-Ŷ|\right|}{N\times C}}$ (14), which is utilized for the subsequent update of the population A(k,G).


(14) $$\begin{array}{l} RMSE_{k,G}=\sqrt{\frac{\left||A_{k,G}W_{k,G}-Ŷ|\right|}{N\times C}} \end{array}$$

In the first iteration, when *G* = 1, we select the individual with the smallest RMSE value as *A*_*best*,1_ and store this RMSE as *RMSE*_*A*_*best*,1__.

#### 3.3.3 Evolutionary operation

Evolutionary operations include mutation and crossover. Different variation strategies are effective in solving various problems. Adaptive evolutionary algorithms can iteratively accumulate experience and select a more suitable variation strategy for mutation operations from the strategy pool based on this experience.

Defined a parameter learning period, denoted as *LP*, which represents a fixed number of iterations for SaE to gain experience. Let *p*_*l,G*_, where *l* = 1, 2, 3, 4, denote the probability that strategy *l* will be selected during the *G*th iteration. The update equation for *p*_*l,G*_ is as follows:


(15) $$\begin{array}{l} p_{l,G}=\{\begin{array}{c} \frac{1}{4},\text{if }G\leq LP\\ \frac{s_{l,G}}{\sum_{l=1}^{4}s_{l,G}},\text{if }G>LP \end{array} \end{array}$$

where


$$s_{l,G}=\frac{\sum_{g=G-LP}^{G}-1ns_{l,g}}{\sum_{g=G-LP}^{G-1}ns_{l,g}+\sum_{g=G-LP}^{G-1}nf_{l,g}}+\varepsilon ,l=1,2,3,4$$

*ns*_*l,g*_ and *nf*_*l,g*_ represent the number of mutation vectors that successfully enter and do not enter the next generation of individual vectors obtained by the mutation strategy *l*, respectively. ε is a small positive value to ensure that it is not null. Then select the mutation strategy in Section 2.3.2 based on the likelihood of each strategy.

For the first *LP* iteration, the probability of fixing each strategy is 0.25 to provide a learning experience to the algorithm. When the number of iterations exceeds *LP*, the *p*_*l,g*_ is re-assigned and the performing strategy has a larger possibility. For the first *LP* iteration, the probability of fixing each strategy is 0.25 to provide a learning experience to the algorithm. When the number of iterations exceeds *LP*, the *p*_*l,g*_ is re-assigned and the performing strategy has a larger possibility by Equation (15).

After generating all mutation vectors, cross-operate each individual vector with its corresponding variation vector using Equation (13).


(16) $$\begin{array}{l} u_{i,G}^{j}=\{\begin{array}{c} v_{i,G}^{j},\text{if }\left(rand_{j}\leq CR\right)\text{ or }\left(j=j_{rand}\right),\\ \theta_{i,G}^{j},\text{otherwise} \end{array} \end{array}$$

In this model, the cross rate *CR* is selected from a normal distribution N(0.5,0.3). The control parameter *F* is selected from a normal distribution N(0.5,0.1). The range of *K* is defined as [0, 1]. In the model, the cross rate *CR* is chosen from a normal distribution with a mean of 0.5 and a standard deviation of 0.3, balancing search diversity and focus. The control parameter *F* is selected from a normal distribution with a mean of 0.5 and a standard deviation of 0.1, controlling mutation intensity. The range of *K* is set between 0 and 1 to balance old and new genetic information during crossover.

#### 3.3.4 Update population

The resulting trail vector is compared with the individual vector of generation *G*. To improve the generalization of the model, the output weight ||*W*|| should be reduced, allowing for better elements to be filtered through Equation (16).


(17) $$\begin{array}{l} A_{k,G+1}^{k}=\{\begin{array}{c} u_{k,G+1},\\ \text{if }RMSE_{A_{k,G}}-RMSE_{u_{k,G+1}}>\epsilon \cdot RMSE_{A_{k,G}}\\ u_{k,G+1},\\ \text{if }|RMSE_{A_{k,G}}-RMSE_{u_{k,G+1}}|<\epsilon \cdot RMSE_{A_{k,G}}\\ \text{and}||W_{u_{k,G+1}}||<||W_{A_{k,G}}||\\ A_{k,G},\text{otherwise} \end{array} \end{array}$$

To determine the population of generation *G*. Where ϵ is a very small positive number, its effect is to introduce a certain level of fault tolerance to the condition.

Continue executing steps 2–4 until either the upper limit of the number of iterations is reached or the best Root Mean Square Error (RMSE) value falls below the predetermined threshold.

Based on the above discussion, we summarized the main steps of our proposed SaE-GBLS models in Algorithm 1.

**Algorithm 1:** SaE-GBLS.

**Input:** training samples *X, Y*, the feature mapping function ϕ(·), the enhance function ξ(·), the number of feature parameters and enhance parameters *n, m*, regularization parameter λ_1_, λ_2_, the number of neighbors *k*_1_, *k*_2_, the learning period *LP* and the population size *NP*
**Output:** output weight *W*
**for** *k* = 1 to NP **do**
Random *A*_*k*,1_
Calculate the corresponding output weight *W*_*k*,1_ and *RMSE*_*k*,1_
**end for**
**for** *i* = 1 to G **do**
for *j* = 1 to NP **do**
Calculate the mutation vector *v*_*j,i*_ by Section 2.3.2
Calculate the trail vector *u*_*j,i*_ by Equation (16)
**end for**
Calculate the output weight Ŵ_*i,j*_ for *u*_*j,i*_ and output weight *W*_*j,i*_ for *A*_*j,i*_ by Equation (12) in Section 2.2
Evaluation the new population by Equation (17)
**end for**

## 4 Experiments

In this chapter, we will apply the SaE-GBLS model to detect epileptic seizures. The chapter consists of an introduction to the datasets and the application of SaE-GBLS for the seizure detection task.

### 4.1 Dataset

**Figure 3:**
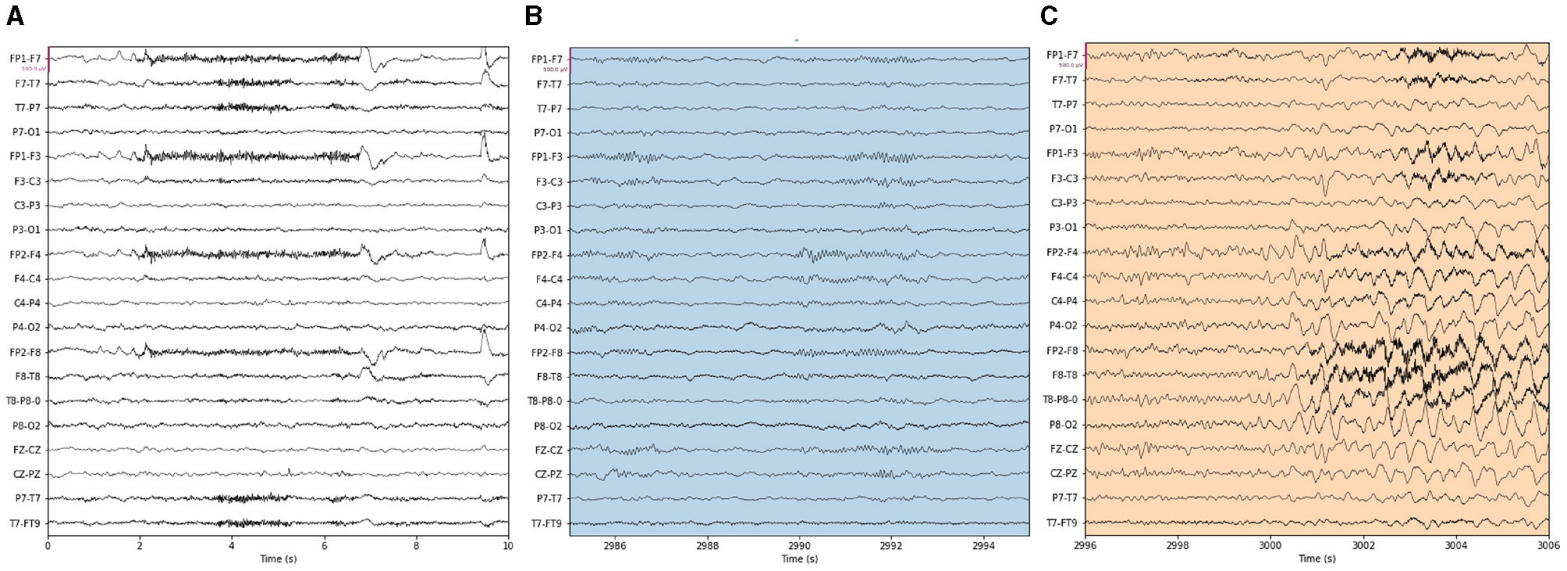
Samples in the CHB-MIT dataset. **(A)** Raw data, **(B)** background, **(C)** seizure.

**Figure 4:**
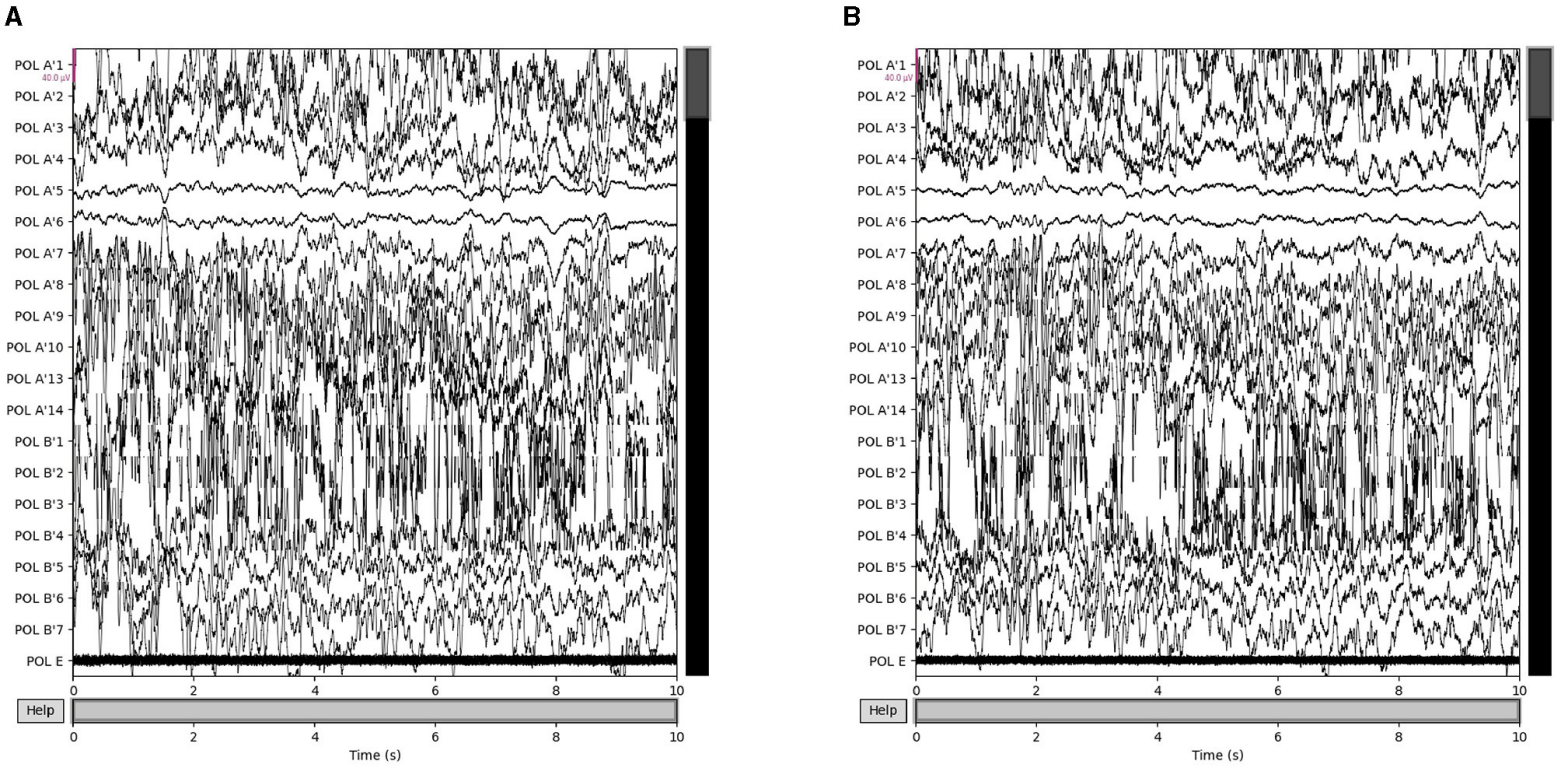
Samples in the Patient dataset. **(A)** Interictal epileptiform discharges and **(B)** epilepsy-like clinical seizure period.

Our proposed approach is used to detect epileptic seizures from EEG. To evaluate the effectiveness of our approach, we employ three publicly accessible datasets: CHB-MIT (Shoeb, 2009), Kaggle (2014), and Bonn (Andrzejak et al., 2001), along with a private dataset named Patient. As for the Bonn dataset, we used the F subset for interictal (non-seizure) periods and the S subset for seizure periods. Some sample signals of CHB-MIT and the patient are shown in Figures 3, 4. The descriptions of these datasets are briefly provided in Table 1.

**Table 1:** Public datasets of epileptic EEG.

**Datasets**	**No.of subjects**	**No. of channels**	**Recording Type**	**Total duration**	**Sampling frequency(Hz)**
CHB-MIT (Shoeb, 2009)	23	23	Scalp	844 h	256
Kaggle (Kaggle, 2014)	2	16	iEEG	627 h	5,000
Bonn (Andrzejak et al., 2001)	10	1	Scalp/iEEG	39 m	173.61
Patient	4	102–179	SEEG	24 h	2,000

#### 4.1.1 Public dataset

**CHB-MIT:** The CHB-MIT Epilepsy EEG dataset, which is publicly accessible on the PhysioNet server, was created through a collaboration between researchers at Children's Hospital Boston (CHB) and the Massachusetts Institute of Technology (MIT). This dataset includes EEG signals from 23 patients with intractable epilepsy, comprising 24 recordings, with two recordings from the same patient. All EEGs in the dataset were collected from children and adolescents and are available in European data format (.edf). The dataset includes multiple seizure and non-seizure recording files for each patient.

**Kaggle:** The dataset consists of intracranial EEG signals obtained from two human subjects and five dogs. As part of the Seizure Detection Challenge organized by the American Epilepsy Society, the human EEG signals were sampled at a frequency of 400 Hz, while the dog EEG signals were sampled at a frequency of 5,000 Hz. Details about the competition and the dataset can be found on the Kaggle website. In this study, we have only used the human dataset.

**Bonn:** The Bonn EEG dataset, which consists of five files labeled A to E, was collected by the Bonn University Epilepsy Institute in Germany. This dataset includes 100 single-channel recordings. Each sequence is 23.6 s long and was sampled at a frequency of 173.61 Hz. The dataset represents the status of normal subjects during different states, including eyes open, eyes closed, epileptogenic focus contralateral, medial, and seizures. Files A and B contain scalp EEG, while files C, D, and E include intracranial EEG.

#### 4.1.2 Private clinical dataset

**Patient:** The collected dataset is Stereoelectroencephalogram (SEEG) data, which uses stereotactic techniques to obtain electrophysiological data from specific brain locations by placing recording electrodes at those locations. The average distance between adjacent electrodes is 2 cm, and it is necessary to record the trajectory of the electrode from the entry point to the target point when placing electrodes in the brain. This dataset contains SEEG data collected from a patient at The Second Affiliated Hospital of Guangzhou University of Chinese Medicine.

Table 1 summarizes the datasets used in our experiments.

### 4.2 Evaluation metrics

To validate the proposed model, the *K*-fold cross-validation technique is employed. The entire dataset is partitioned into five equal-sized folds, where 1-fold is designated as the test set, and the remaining 4-folds constitute the training sets. Across all experiments, 80% of the samples were allocated for training, while the remaining 20% were designated for testing. The assessment of classification performance involved the use of metrics such as accuracy, precision, sensitivity, and F1-score. Calculations for all metrics were conducted using a default threshold value of 0.5. It is important to note that the threshold value can significantly impact the classification performance, particularly in scenarios with class imbalance or varying costs associated with false positives and false negatives. A higher threshold would prioritize precision, reducing false alarms at the potential cost of missing some true seizure events, while a lower threshold would favor recall, increasing seizure detection sensitivity but potentially increasing false positive rates. The choice of retaining the default threshold of 0.5 was primarily motivated by the exploratory nature of this work and the desire to establish a baseline performance without introducing additional complexity through threshold tuning. The evaluation metrics, including accuracy, precision, recall (sensitivity), and F1-score, as shown in Equations (18–21):


(18) $$\begin{array}{l} Accuracy=\frac{TP+TN}{TP+TN+FP+FN} \end{array}$$

(19) $$\begin{array}{l} Sensitivity=Recall=\frac{TP}{TP+FN} \end{array}$$

(20) $$\begin{array}{l} Precision=\frac{TP}{TP+FP} \end{array}$$

(21) $$\begin{array}{l} F1-score=\frac{2*Precision*Recall}{Precision+Recall} \end{array}$$

Here, the *TP* represents the number of correctly predicted seizure segments, while the *FN* indicates the number of seizure segments that were mistakenly classified as non-seizure segments. Likewise, the *TN* represents the number of correctly classified no-seizure segments, while the *FP* represents the number of no-seizure segments that were mistakenly identified as seizure segments. The *F*1 − *score* provides a comprehensive evaluation of the model's capability to accurately identify instances in unbalanced datasets by considering both accuracy and recall. Accuracy provides an overall measure of the model's performance in correctly classifying both seizure and non-seizure segments. However, in the clinical domain of epilepsy monitoring, the consequences of misclassification can be severe, necessitating a more nuanced assessment beyond mere accuracy. Precision quantifies the model's ability to avoid false positives, which correspond to falsely identifying non-seizure segments as seizures. High precision is crucial in this context, as false alarms can lead to unnecessary interventions, patient anxiety, and potential overmedication. Recall, on the other hand, reflects the model's capability to detect true positive cases, i.e., correctly identifying seizure segments. High recall is essential to ensure that no actual seizure events are missed, as missed detections can have grave implications for patient safety and timely treatment. The F1-score, being the harmonic mean of precision and recall, provides a balanced assessment by capturing both the model's ability to minimize false positives and its ability to identify true positives effectively. This composite metric is particularly valuable in the epilepsy detection context, where both precision and recall are equally critical. By evaluating the model's performance using these metrics, a comprehensive understanding of its strengths and weaknesses can be obtained.

### 4.3 Experimental setting

We compared our approach against various classifiers commonly used in the field of electroencephalography signal classification, including K-Nearest Neighbors (KNN) (Amin et al., 2015; Shih et al., 2022), Decision Tree(DT) (Siddiqui et al., 2019; Shih et al., 2022), Random Forest(RF) (Sharma et al., 2018; Siddiqui et al., 2019), Adaboost, Gaussian Naive Bayes (GaussianNB) (Shih et al., 2022), EEGNet (Lawhern et al., 2018), Long Short-Term Memory (LSTM), Gated Recurrent Unit (GRU), BLS and GBLS. We use the same data preprocessing and data input for all of the methods. The epochs in the deep learning methods are all set to 100. In addition, for the publicly available datasets CHB-MIT, Kaggle, and Bonn we have cited results from other papers for comparison, including Support Vector Machine(SVM) (Ein Shoka et al., 2021), Ensemble (Usman et al., 2021), and ANFIS-PSO (Shoeibi et al., 2022).**KNN:** Using the class labels of the K nearest labeled samples, an income-based learning method can identify the class label of an unlabeled sample by finding the K nearest labeled samples in the training set.**DT:** The goal of data screening and decision-making is achieved by constructing a mathematical model based on the characteristics of the data and utilizing the concept of classification.**RF:** Random Forest is a decision tree-based ensemble learning technique that combines multiple decision trees. Every time, a random selection of features is used as input, and the data collection is chosen randomly with replacement.**Adaboost:** Adaboost is a machine learning technique that combines several basic classifiers based on their error rate. The overall classification accuracy is significantly improved by linearly combining multiple weak classifiers and then voting based on the weight of each classifier.**GaussianNB:** This is one of the Naive Bayes algorithms.**EEGNet:** mIt is a deep learning architecture specifically designed for the analysis of electroencephalography (EEG) data. It can accurately classify EEG signals with high temporal resolution and robustness to noise.

**LSTM:** The Long Short-Term Memory (LSTM) model, which is a type of recurrent neural network (RNN), is known for its ability to effectively capture long-range dependencies and patterns in sequential data. Its unique architecture includes memory cells and gating mechanisms, enabling it to retain information for extended periods. LSTMs are widely used in various fields, especially in natural language processing and time series analysis, because of their ability to effectively model sequences and address the issue of vanishing gradients.**GRU:** The Gated Recurrent Unit (GRU) model is a variant of recurrent neural network (RNN) that is specifically designed for processing sequential data. GRUs are characterized by their simplified architecture compared to traditional RNNs and LSTMs. They feature gating mechanisms that regulate the flow of information through the network, enabling the capture of long-term dependencies while addressing vanishing gradient issues. GRUs have gained prominence in various applications, particularly in natural language processing and time series analysis, due to their computational efficiency and competitive performance in modeling sequential data.**BLS:** The BLS architecture first randomly maps the input into multiple feature spaces which comprise the feature nodes. The outputs of these feature nodes are then expanded extensively to constitute the enhancement nodes. Finally, the output weights of the network are analytically computed based on the mappings through the feature and enhancement nodes.**GBLS:** The GBLS takes into account the local invariance of the data and incorporates stream shape learning into the objective function of the standard BLS.**SVM:** SVMs are supervised learning models that find the optimal hyperplane that maximizes the margin between classes in the feature space, utilizing support vectors which are the data points closest to the decision boundary.**Ensemble:** An ensemble classifier that combines the output of SVM, CNN, and LSTM using model agnostic meta learning.**ANFIS-PSO:** A method of combining fuzzy theory and deep learning techniques and introducing particle swarm optimization algorithm for optimization.

### 4.4 Experimental results

Tables 2–5 present a comparison between our proposed approach and previous methods for the seizure detection task. The results of the 5-fold cross-validation are shown in Tables 6–9. We chose the best 1-fold results to compare with other models. In comparison to traditional machine learning methods, SaE-GBLS achieves the highest accuracy, precision, sensitivity, and F1-score across all four datasets. In comparison to deep learning, it is clear that both LSTM and SaE-GBLS exhibit excellent performance, on the CHB-MIT dataset, LSTM outperforms SaE-GBLS overall, suggesting that LSTM does have greater potential for time-series data, but SaE-GBLS also shows comparable results, as shown in Table 2.

**Table 2:** Comparison between SaE-GBLS and other approaches on CHB-MIT dataset.

**Methods**	**Accuracy**	**Precision**	**Sensitivity**	**F1-score**
KNN	0.84740	0.92466	0.75870	0.83350
DT	0.86584	0.88621	0.84158	0.86331
RF	0.88566	0.91700	0.84982	0.88212
Adaboost	0.84463	0.86055	0.82509	0.84245
GassianNB	0.67450	0.84281	0.43452	0.57341
EEGNet	0.87252	0.89226	0.84936	0.87028
LSTM	**0.92831**	**0.93782**	0.91850	**0.92806**
GRU	0.90572	0.91453	0.89652	0.90543
BLS	0.88589	0.87092	0.89707	0.88380
GBLS	0.88313	0.86544	0.90733	0.88589
SVM	0.85000	0.82979	0.90698	0.86667
SaE-GBLS (ours)	0.92358	0.91357	**0.93568**	0.92450

Bold values indicate the best performance for each metric.

**Table 3:** Comparison between SaE-GBLS and other approaches on the Kaggle dataset.

**Methods**	**Accuracy**	**Precision**	**Sensitivity**	**F1-score**
KNN	0.69978	0.65860	0.85360	0.74353
DT	0.89506	0.93281	0.85581	0.89265
RF	0.83418	**0.99837**	0.67584	0.80604
Adaboost	0.95202	0.95722	0.94827	0.95272
GassianNB	0.81145	0.99057	0.63621	0.77480
EEGNet	0.93547	0.91135	0.96753	0.93860
LSTM	0.79377	0.80190	0.79086	0.79634
GRU	0.80415	0.82248	0.78536	0.80349
BLS	0.91759	0.87194	0.95661	0.91232
GBLS	0.93426	0.92792	0.94167	0.93474
Ensemble	0.95530	–	0.94200	–
SaE-GBLS (ours)	**0.99259**	0.99077	**0.99444**	**0.99261**

Bold values indicate the best performance for each metric.

**Table 4:** Comparison between SaE-GBLS and other approaches on the Bonn dataset.

**Methods**	**Accuracy**	**Precision**	**Sensitivity**	**F1-score**
KNN	0.92424	0.93750	0.90909	0.92308
DT	0.93939	0.96774	0.90909	0.93750
RF	0.93939	0.93939	0.93939	0.93939
Adaboost	0.98485	0.97059	**1.00000**	0.98507
GassianNB	0.83333	0.92308	0.72727	0.81356
EEGNet	0.87879	0.93103	0.81818	0.87097
LSTM	0.87879	0.90323	0.84848	0.87500
GRU	0.87879	0.82051	0.96970	0.88889
BLS	0.97500	**1.00000**	0.95238	0.97561
GBLS	0.97500	**1.00000**	0.95000	0.97436
ANFIS-PSO	0.99790	0.99420	0.99830	0.99810
SaE-GBLS (ours)	**1.00000**	**1.00000**	**1.00000**	**1.00000**

Bold values indicate the best performance for each metric.

**Table 5:** Comparison between SaE-GBLS and other approaches on the Patient dataset.

**Methods**	**Accuracy**	**Precision**	**Sensitivity**	**F1-score**
KNN	0.91044	0.96471	0.84828	0.90275
DT	0.97169	0.97813	0.96379	0.97091
RF	0.96113	0.98370	0.93621	0.95936
Adaboost	0.97803	0.98427	0.97069	0.97743
GassianNB	0.64385	0.91384	0.30172	0.45366
EEGNet	0.97676	0.97263	0.98017	0.97638
LSTM	0.99606	0.99209	**1.00000**	0.99603
GRU	0.97830	0.97619	0.98008	0.97813
BLS	0.95371	0.93631	0.96966	0.95269
GBLS	0.96134	0.95116	0.97375	0.96232
SaE-GBLS (ours)	**0.99810**	**0.99626**	**1.00000**	**0.99813**

Bold values indicate the best performance for each metric.

**Table 6:** The 5-fold cross-validation results of SaE-GBLS on the CHB-MIT dataset.

**Fold**	**Accuracy**	**Precision**	**Sensitivity**	**F1-score**
Fold1	0.92358	0.91357	0.93568	0.9245
Fold2	0.91286	0.89853	0.93084	0.9144
Fold3	0.91909	0.90891	0.93154	0.92008
Fold4	0.91978	0.91293	0.92808	0.92044
Fold5	0.91217	0.90108	0.92600	0.91337

**Table 7:** The 5-fold cross-validation results of SaE-GBLS on the Kaggle dataset.

**Fold**	**Accuracy**	**Precision**	**Sensitivity**	**F1-score**
Fold1	0.99259	0.99077	0.99444	0.99261
Fold2	0.98796	0.98349	0.99259	0.98802
Fold3	0.99259	0.98987	0.99537	0.99261
Fold4	0.99583	0.99537	0.99630	0.99584
Fold5	0.98981	0.98710	0.99259	0.98984

**Table 8:** The 5-fold cross-validation results of SaE-GBLS on the Bonn dataset.

**Fold**	**Accuracy**	**Precision**	**Sensitivity**	**F1-score**
Fold1	1.00000	1.00000	1.00000	1.00000
Fold2	1.00000	1.00000	1.00000	1.00000
Fold3	1.00000	1.00000	1.00000	1.00000
Fold4	1.00000	1.00000	1.00000	1.00000
Fold5	1.00000	1.00000	1.00000	1.00000

**Table 9:** The 5-fold cross-validation results of SaE-GBLS on the Patient dataset.

**Fold**	**Accuracy**	**Precision**	**Sensitivity**	**F1-score**
Fold1	0.99810	0.99626	1.00000	0.99813
Fold2	0.99746	0.99502	1.00000	0.99750
Fold3	0.99430	0.99253	0.99625	0.99439
Fold4	0.99810	0.99750	0.99875	0.99812
Fold5	0.99556	0.99133	1.00000	0.99564

As for the Kaggle dataset, Table 3 displays the results. In Table 3, SaE-GBLS achieves a 5.712% improvement in accuracy, 7.942% in precision, 2.691% in sensitivity, and 5.401% in F1-score compared to EEGNet.

And in Table 4, we can observe that SaE-GBLS outperforms EEGNet, LSTM, and GRU by a significant margin in all four metrics and achieves a perfect score of 1. In addition to SaE-GBLS, GBLS and BLS also achieved good performance. The small size of the Bonn dataset, which consists of only one sampling channel and relatively simple data, could contribute to this phenomenon. On the other hand, EEGNet, LSTM, and GRU are deep neural networks that may lead to overfitting. Meanwhile, SaE-GBLS inherits the advantages of BLS and has a simple structure, which makes it less likely to overfit even with small data and can yield superior outcomes.

Table 5 shows the results of the actual dataset named “Patient”. From Table 5, we can see that both LSTM and SaE-GBLS excel, but the latter performs more consistently. All in all, SaE-GBLS demonstrates the most consistent performance across the four datasets. Furthermore, other methods also demonstrate satisfactory performance on this dataset. To some extent, we can suggest that the data we collected is of good quality.

We can observe that deep learning methods and the integrated learning method Adaboost outperform other machine learning methods. However, on the Bonn dataset, which contains a small amount of data, EEGNet, LSTM, and GRU tend to overfit. On the other hand, our proposed SaE-GBLS performs well on all four datasets, surpassing the other methods overall. As can be seen from Tables 2, 4, SaE-GBLS achieves the highest values in all evaluation metrics on the CHB-MIT and Bonn datasets. Additionally, it achieves the highest accuracy, precision, and F1-score on the Kaggle dataset (see Table 3). In general, our method can effectively detect the onset of epileptic seizures.

### 4.5 Discussion

To date, extensive research has been conducted on the diagnosis of epileptic seizures using artificial intelligence techniques. Due to the presence of complex structures, deep learning models excel at extracting the abstract and underlying characteristics of data. As a result, they have gained widespread popularity and are favored by numerous researchers. In a study conducted by the authors in Hossain et al. (2019), during their preliminary studies on diagnosing epileptic seizures, Taqi et al. (2017) introduced a novel 2D-CNN model that aimed to extract spectrum and time features from EEG recordings. This model was specifically designed to capture the overall structure of seizures. When applied to the Bern-Barcelona dataset, the model successfully extracted features and achieved remarkable results. However, it is worth noting that deep learning methods typically require a significant amount of data for training and consume a considerable amount of training time.

Our research is based on BLS, and because of BLS's ability to learn incrementally, our model can be updated dynamically when new data is added, eliminating the need for retraining. It is undeniable that BLS has certain limitations in terms of feature extraction and may not perform as well as deep learning methods. Firstly, the random mapping feature layer in BLS, although endowing the network with some nonlinear mapping capability, may have relatively limited feature extraction power compared to deep neural networks. Deep models can automatically learn hierarchical feature representations through multiple nonlinear transformations, ranging from low-level edges and textures to high-level semantic abstractions. Moreover, BLS's feature extraction process heavily relies on the generation of random mapping matrices, making it sensitive to changes in data distribution and quality. If the input data distribution undergoes significant shifts, the current random mapping matrices may fail to effectively capture the features of the new distribution, thereby impacting model performance. In contrast, deep learning models, through end-to-end training, exhibit a certain degree of adaptability to input distribution changes. There is a wide range of methods used for detecting epileptic seizures, but none of them can be definitively considered superior to others. The optimal choice of structure for seizure detection should be made with careful consideration of the dataset and the specific characteristics of the problem.

Although our approach has shown commendable effectiveness across the four datasets we have used, it is still hindered by certain limitations. For example, there is significant potential for enhancement in the design of the feature extraction module, and integrating this method into real-world clinical medical scenarios remains a challenging task. One potential limitation of the current feature extraction approach lies in its reliance solely on statistical features derived from the EEG signals. While these handcrafted features capture essential statistical properties, they may fail to fully characterize the intricate spatio-temporal patterns and nonlinear dynamics inherent in the epileptic brain activity. To address this limitation, future work could explore the incorporation of more sophisticated feature extraction techniques. First, connectivity measures quantifying the functional relationships between different brain regions, such as coherence and phase-locking values, could be incorporated. These features may provide insights into the propagation patterns of epileptic activity across the brain. Furthermore, the application of deep learning techniques for automated feature extraction could be investigated. Convolutional neural networks (CNNs) and recurrent neural networks (RNNs) have demonstrated remarkable ability to learn hierarchical representations directly from raw signal data, potentially capturing intricate spatio-temporal patterns that may be overlooked by handcrafted features. It is worth noting that the adoption of deep learning approaches may require larger datasets and computational resources, but could potentially lead to more robust and generalizable models by leveraging the ability of these methods to learn discriminative features directly from the data. It is our goal to continuously improve and implement it in real medical situations in our forthcoming endeavors.

## 5 Conclusion

In this paper, we introduce a novel graph-broad ensemble learning system (SaE-GBLS) for the detection of epileptic seizures from electroencephalographic (EEG) data. The key contributions of this work lie in the innovative integration of a self-adaptive evolutionary algorithm for network pruning, the incorporation of geometric and judgment information during the training process, and the optimization of feature and enhancement node counts to mitigate the impact of non-optimal nodes. Compared to other methods, our proposed SaE-GBLS model demonstrated superior or comparable performance in detecting epileptic seizures across three public datasets and one actual clinical dataset. Specifically, SaE-GBLS achieved an average F1-score of 0.97 over the four datasets, while also maintaining a high sensitivity of 0.94 and a high precision of 0.91. These results indicate the effectiveness of our model in minimizing both missed detections and false alarms. Despite these promising results, certain limitations should be acknowledged. The current feature extraction module relies primarily on statistical features derived from the EEG signals, potentially failing to capture more complex spatio-temporal patterns and nonlinear dynamics inherent in epileptic brain activity. To address this issue, future work will focus on enhancing the feature extraction capabilities by exploring frequency-domain features, connectivity measures, and deep learning techniques for automated feature learning. The proposed SaE-GBLS model holds significant potential for real-world clinical applications, offering a reliable and efficient tool for assisting doctors to detect epileptic events in a timely and accurate manner greatly reduces their labor costs and promotes early intervention.

## Data availability statement

The original contributions presented in the study are included in the article/supplementary material, further inquiries can be directed to the corresponding authors.

## Ethics statement

The studies involving humans were approved by Ethics Committee of Guangzhou Provincial Hospital of Traditional Chinese Medicine (Approval No. DF2021-030-01). The studies were conducted in accordance with the local legislation and institutional requirements. The participants provided their written informed consent to participate in this study.

## Author contributions

LC: Writing – review & editing, Writing – original draft, Visualization, Validation, Supervision, Project administration, Methodology, Data curation, Conceptualization. JX: Writing – original draft, Validation, Software, Methodology, Data curation. JD: Writing – original draft, Project administration, Methodology, Supervision, Resources, Funding acquisition, Conceptualization, Writing – review & editing. YZ: Validation, Software, Writing – review & editing. CC: Writing – review & editing, Visualization. JZ: Writing – review & editing, Supervision, Resources, Conceptualization. ZZ: Writing – review & editing, Visualization. YQ: Writing – review & editing, Supervision, Resources, Conceptualization.
